# Supplementary material for: Development of a camera trap for perching dragonflies: a new tool for freshwater environmental assessment
Source: PeerJ. 2020 Sep 18;8:e9681. doi: 10.7717/peerj.9681 (PMC7505062; doi:10.7717/peerj.9681)
Supplement: Supplemental Information 1 — The two CDS resistors (CDS cells) correspond to the two light sensors in the detector section in Fig. 1. The ”Det.” CDS cell and the ”Ref.” CDS cell correspond to the first (upper) sensor and the second sensor, respectively. [file peerj-08-9681-s001.pdf]

Yoshioka et al. Development of a camera trap for perching dragonflies: a new tool for freshwater environmental assessment.

Supplemental Fig. S1

Circuit diagram for the dragonfly detector

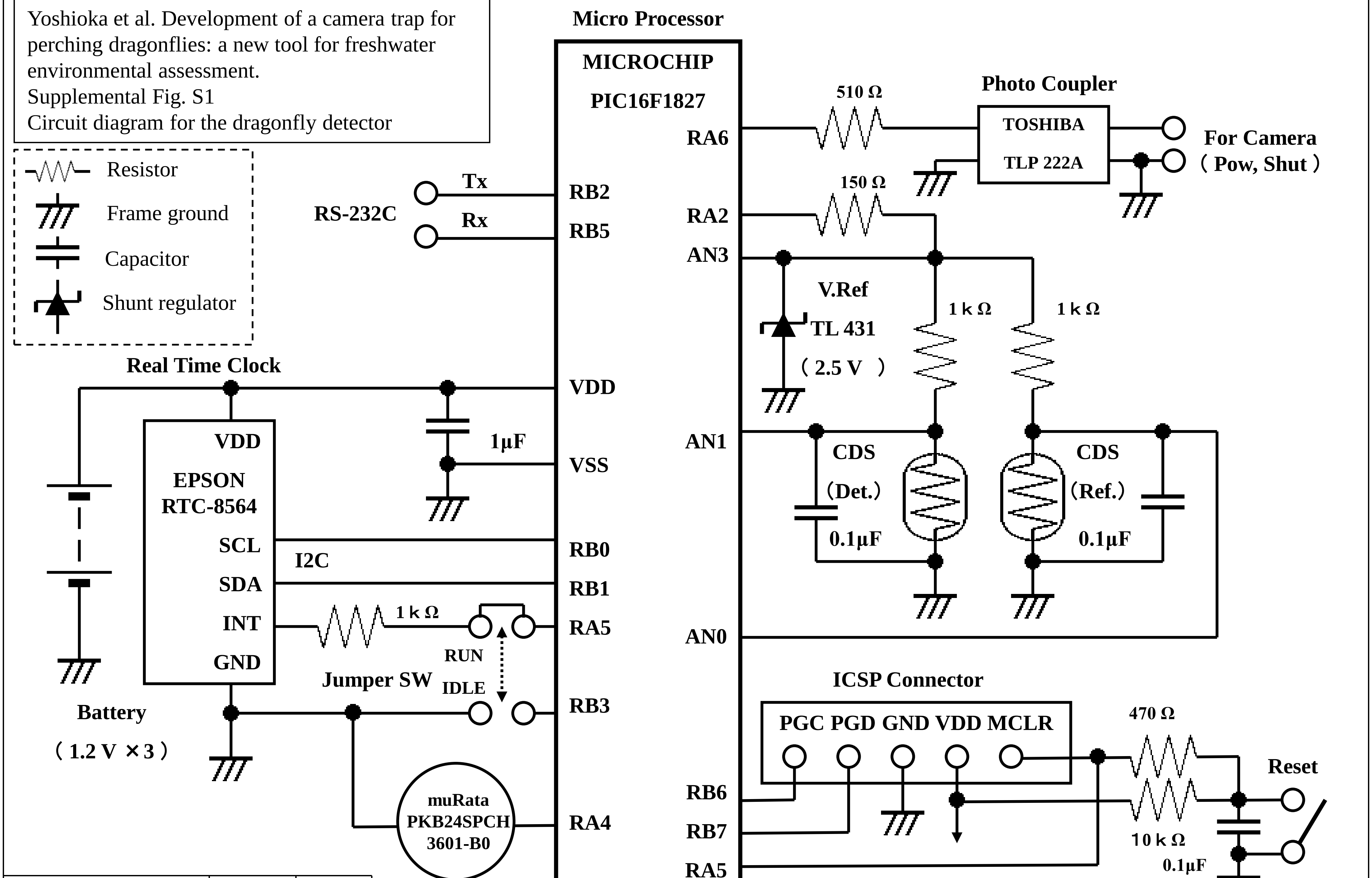

Dragonfly Detector	8/28/16	S.A.
